# Deconvolution of B cell receptor repertoire in multiple sclerosis patients revealed a delay in tBreg maturation

**DOI:** 10.3389/fimmu.2022.803229

**Published:** 2022-08-16

**Authors:** Yakov A. Lomakin, Ivan V. Zvyagin, Leyla A. Ovchinnikova, Marsel R. Kabilov, Dmitriy B. Staroverov, Artem Mikelov, Alexey E. Tupikin, Maria Y. Zakharova, Nadezda A. Bykova, Vera S. Mukhina, Alexander V. Favorov, Maria Ivanova, Taras Simaniv, Yury P. Rubtsov, Dmitriy M. Chudakov, Maria N. Zakharova, Sergey N. Illarioshkin, Alexey A. Belogurov, Alexander G. Gabibov

**Affiliations:** ^1^ Shemyakin-Ovchinnikov Institute of Bioorganic Chemistry Russian Academy of Sciences (RAS), Moscow, Russia; ^2^ Institute of Chemical Biology and Fundamental Medicine, Siberian Branch Russian Academy of Sciences (RAS), Novosibirsk, Russia; ^3^ Skolkovo Institute of Science and Technology, Moscow, Russia; ^4^ Department of Molecular Technologies, Institute of Translational Medicine, Pirogov Russian National Research Medical University, Moscow, Russia; ^5^ Institute for Information Transmission Problems (Kharkevich Institute), Russian Academy of Sciences (RAS), Moscow, Russia; ^6^ Vavilov Institute of General Genetics, Russian Academy of Sciences (RAS), Moscow, Russia; ^7^ Quantitative Sciences Division, Department of Oncology, Johns Hopkins University, Baltimore, MD, United States; ^8^ Neuroinfection Department of the Research Center of Neurology, Moscow, Russia; ^9^ Department of Biological Chemistry, Evdokimov Moscow State University of Medicine and Dentistry, Moscow, Russia; ^10^ Department of Life Sciences, Higher School of Economics, Moscow, Russia; ^11^ Department of Chemistry, Lomonosov Moscow State University, Moscow, Russia

**Keywords:** B regulatory cells, BCR, CD19+CD24highCD38high, multiple sclerosis, MS, transitional Breg, TrB, activated memory-like transitional cells

## Abstract

**Background:**

B lymphocytes play a pivotal regulatory role in the development of the immune response. It was previously shown that deficiency in B regulatory cells (Bregs) or a decrease in their anti-inflammatory activity can lead to immunological dysfunctions. However, the exact mechanisms of Bregs development and functioning are only partially resolved. For instance, only a little is known about the structure of their B cell receptor (BCR) repertoires in autoimmune disorders, including multiple sclerosis (MS), a severe neuroinflammatory disease with a yet unknown etiology. Here, we elucidate specific properties of B regulatory cells in MS.

**Methods:**

We performed a prospective study of the transitional Breg (tBreg) subpopulations with the CD19^+^CD24^high^CD38^high^ phenotype from MS patients and healthy donors by (i) measuring their content during two diverging courses of relapsing-remitting MS: benign multiple sclerosis (BMS) and highly active multiple sclerosis (HAMS); (ii) analyzing BCR repertoires of circulating B cells by high-throughput sequencing; and (iii) measuring the percentage of CD27^+^ cells in tBregs.

**Results:**

The tBregs from HAMS patients carry the heavy chain with a lower amount of hypermutations than tBregs from healthy donors. The percentage of transitional CD24^high^CD38^high^ B cells is elevated, whereas the frequency of differentiated CD27^+^ cells in this transitional B cell subset was decreased in the MS patients as compared with healthy donors.

**Conclusions:**

Impaired maturation of regulatory B cells is associated with MS progression.

## Introduction

Multiple sclerosis is a highly heterogenous severe autoimmune neurodegenerative disorder with an evident inflammation component (1). Despite considerable advances in this field, the mechanism triggering it remains elusive, hindering the development of effective therapeutics (2–8). MS progression is mostly associated with the promotion of the T cell response (9, 10). Yet the contribution of B cells to various autoimmune disorders, including MS, should not be underestimated (11–16). Apart from antibody production and antigen presentation, B cells play a crucial role in regulating the immune response through their antibody-independent effector functions (17, 18).

In 1974, professor James Turk and coauthors suggested that B cells could inhibit inflammation during delayed hypersensitivity reactions (19). Further on, regulatory properties of B cells were confirmed in experimental autoimmune encephalomyelitis (EAE), an animal model of MS (20). The subpopulation of B cells performing regulatory functions were termed Bregs. In mice, the Breg population constitutes up to 5% of total B cells in the spleen and lymph nodes, and their number significantly increases during inflammation development (21–23). In humans, Bregs account for less than 5% of blood B cells (24). Abnormalities in the Breg counts or their function were observed in patients with autoimmune diseases (25–29) and allergies (30).

Different phenotypes of Bregs are described so far. Transitional Bregs (tBreg cells) CD19^+^CD24^high^CD38^high^ (24, 26, 29, 31) and memory Bregs CD19^+^CD24^high^CD27^+^ are the most studied regulatory B cell subpopulations that modulate the immune response in humans (32, 33). Meanwhile, several other Breg phenotypes are reported: CD19^+^CD25^+^CD71^+^CD73^-^ (34), CD19^+^CD27^int^CD38^+^IgM^+^ (35), CD19^+^CD24^high^CD27^+^CD39^high^IgD^-^IgM^+^CD1c^+^ (36), CD19^+^CD5^+^Foxp3^+^ (37), and CD19^+^CD38^+^CD1d^+^IgM^+^CD147^+^ (38). Transitional CD19^+^CD24^high^CD38^high^ Bregs can be found in the peripheral blood of healthy adults representing a minor subset (approximately 4%) of all circulating B cells (39). This tBreg subset was previously shown to produce IL-10, regulate CD4^+^ T cell proliferation/differentiation toward T helper effector cells (40), and contribute to the cytokine imbalance during autoimmune diseases (41).

Since the molecular underpinnings of MS onset and progression were revealed, T cell–mediated immunity was believed to play the leading role in it. However, it is evident now that B cells are crucial for MS pathogenesis as well (42, 43). Autoreactive B cells in MS may produce catalytic antibodies, hydrolyzing myelin basic protein (13, 44, 45), and cause humoral cross-reactivity between myelin and viral antigens (46–50). Still, the existing studies on Bregs functioning in MS are controversial and far from conclusive. The percentages of IL-10–producing Bregs in MS patients are shown to be lower than in healthy controls (28, 51). Other works report either unaltered (52, 53) or even increased (25, 54) Breg numbers during MS progression. A recent study shows no association between the reduced peripheral blood Bregs levels and the Expanded Disability Status Scale (EDSS) score in MS (51).

These inconsistencies most likely arise from several Breg populations coexisting (55, 56). Indeed, these regulatory subpopulations can comprise B cells at different stages of development; therefore, the level of certain BCR hypermutations can be lower at the earlier stages of maturation of these B cells. The specificity of Bregs’ BCRs and pathways that mediate their maturation are still poorly elucidated. It is still not known whether the specificity of Bregs BCRs undergoes alteration in autoimmune disorders.

This study aims to identify the possible abnormalities in the structure of BCRs in the tBregs isolated from peripheral blood of MS patients as compared with HD. Thus, we suggest that analyzing BCR repertoires of tBregs may reveal the disease-related alterations occurring at the early stage of B cell development.

## Materials and methods

### Patients and healthy donors

Peripheral blood was obtained from the Neuroinfection Department of the Research Center of Neurology, Moscow, Russia. Venous blood was collected in EDTA Vacutainers (BD) from 19 MS patients (nine with BMS (57) and 10 with HAMS) (58) and 16 HD (**Table 1**). The age of the MS patients ranged between 23 and 70 years old. Their EDSS scores ranged between 1.5 and 8.5. The EDSS values (scored on a scale of 0 to 10) were calculated based on the Kurtzke EDSS scale (59). BMS was diagnosed if the EDSS score was less than 4 for at least 10 years after the disease onset in the absence of treatment; HAMS relapsing-remitting MS was diagnosed based on an EDSS score of 4.0 within five years after the disease onset, poor response to disease-modifying treatments, and two or more relapses with incomplete recovery during one year. None of the patients received glucocorticoid treatment or immunomodulatory treatment for at least six months prior to blood collection. Data on the course of the disease, its duration, and history of administration of disease-modifying treatments are presented in **Table 1**. HDs (24–68 years old) had neither autoimmune nor oncology diseases nor recent infections. The study was approved by the Local Ethics Committee of the Research Center of Neurology and was conducted in full compliance with the WMA Declaration of Helsinki, ICH GCP, and appropriate local legislation. All patients provided written informed consent for enrollment, followed by a discussion of the study with the investigators.

**Table 1:** Baseline and clinical characteristics of patients with multiple sclerosis and healthy donors.

MS phenotype	Age, years	Gender	EDSS	Treatment	Disease duration, years	BCR repertoire analysis	CD27^+^ phenotypic analysis
BMS	56	female	2.5	No treatment	11	+	–
BMS	61	female	3	No treatment	26	+	–
BMS	43	female	1.5	No treatment	12	+	–
BMS	36	male	2.5	No treatment	14	+	–
BMS	46	female	2	No treatment	27	–	+
BMS	43	female	2.5	No treatment	27	–	+
BMS	43	male	4.0	No treatment	18	–	+
BMS	58	female	3.5	No treatment	30	–	+
BMS	70	female	4.0	No treatment	30	–	+
HAMS	33	male	6	IFNβ1b (2006-2011; 2014-2017)	12	+	–
HAMS	23	male	5	No treatment	3	+	–
HAMS	37	female	5	IFNβ1b (2014-2016) GA (2016-2017)	5	+	–
HAMS	29	female	8	GA (2012-2014) IVIG (2014) IFNβ1b (2015-2016)	12	+	–
HAMS	39	female	8.5	No treatment	8	+	–
HAMS	22	female	4.5	No treatment	1	–	+
HAMS	46	male	6	IFNβ1b (2019)	2	–	+
HAMS	44	male	8.5	IFNβ1b (2014-2015)	13	–	+
HAMS	24	male	4.0	No treatment	2	–	+
HAMS	44	female	4.5	No treatment	2	–	+
Healthy	24	female	N/A	N/A	N/A	+	–
Healthy	40	female	N/A	N/A	N/A	+	–
Healthy	36	male	N/A	N/A	N/A	+	–
Healthy	27	female	N/A	N/A	N/A	+	–
Healthy	42	female	N/A	N/A	N/A	+	–
Healthy	25	female	N/A	N/A	N/A	+	–
Healthy	39	male	N/A	N/A	N/A	–	+
Healthy	42	female	N/A	N/A	N/A	–	+
Healthy	35	female	N/A	N/A	N/A	–	+
Healthy	51	female	N/A	N/A	N/A	–	+
Healthy	24	female	N/A	N/A	N/A	–	+
Healthy	34	male	N/A	N/A	N/A	–	–
Healthy	24	female	N/A	N/A	N/A	–	–
Healthy	35	male	N/A	N/A	N/A	–	+
Healthy	68	female	N/A	N/A	N/A	–	+
Healthy	47	male	N/A	N/A	N/A	–	+

IFNβ1b,  interferon-β-1b; GA,  glatiramer acetate; IVIG, intravenous immunoglobulin; HAMS, highly active MS; BMS, benign MS; HD, healthy donors; N/A, not applicable.“+” indicates that the corresponding analysis has been carried out.“-” indicates that the corresponding analysis has not been performed.

### FACS sorting of tBreg and total B cell subsets

Blood samples were diluted two times in PBS with 2 mM EDTA and layered onto Ficoll–Paque Plus (GE Healthcare) and then centrifuged at 900 g for 40 minutes at room temperature. PBMC isolated from MS patients were incubated with ACK lysing buffer for complete removal of red blood cells. Cells were washed with PBS, incubated with α-CD19-PE-Cy7, α-CD24-PE, α-CD38-APC, α-CD27-FITC, and α-CD45-APC-Cy7 antibodies (Biolegend, USA) or α-CD19-PE-Cy7, α-CD24-PE, α-CD38-APC, α-CD45-APC-Cy7, and sytox green dead cell stain (ThermoFisher Scientific) for 60 minutes at +4°C in the dark. Human Fc-blocker (Miltenyi Biotec) was added to all samples before cell staining. B cell subsets were identified by the following markers on their surface: transitional Bregs (CD19^+^CD24^high^CD38^high^), T1 transitional cells (CD19^+^CD24^+++^CD38^+++^), T2 transitional cells (CD19^+^CD24^++^CD38^++^), memory Bregs (CD19^+^CD24^high^CD27^+^), and memory (CD19^+^CD24^+/high^CD38^+/low^CD27^+^) or naïve (CD19^+^CD24^+^CD38^+/low^CD27^-^) peripheral B cells (60). Following incubation, the cells were washed with PBS and resuspended in PBS. To distinguish T1 and T2 cells by CD24 and CD38 expression, we used gates for flow cytometry analysis, which provided a T2/T1 ratio of approximately 3:1 in HDs (61). Leukocyte and total lymphocyte counts per mm^3^ were determined using a hematology analyzer (Nihon Kohden MEK-7222, Nihon Kohden, Japan). All samples were analyzed for B cell and tBreg frequencies by flow cytometry. For nine MS patients (four with BMS and five with HAMS) and six healthy donors, a live CD19^+^ pool of total B cells or CD19^+^CD24^high^CD38^high^ tBreg was sorted into two distinct populations (**Table 1**). The cells were sorted directly into 1.5-mL microcentrifuge tubes containing Qiazol lysis reagent (Qiagen, Germany). Sorting was carried out using a BD FACSAria III, and the data were analyzed using FlowJo software 9.7.5 (TreeStar, Ashland, OR, USA).

### Library preparation for immunoglobulin sequencing (RT-PCR)

RNA extraction was performed using the RNeasy Mini Kit (Qiagen, Germany) according to the manufacturer’s protocol. Reverse transcription (RT) was performed in 20 µL reaction volume using MMLV RT according to the manufacturer’s protocol (Evrogen, Russian Federation). Multiplex PCR with a modified set of the previously described VH- and VL-specific primers was used for cDNA amplification (62). The primers included 15 human VH-specific forward primers and four human JH-specific reverse primers for the IGH chain, 13 human Vκ-forward primers and two Jκ-reverse primers for Vκ genes, and 16 human Vλ-forward primers and three Jλ-reverse primers for Vλ genes (**Table S1**). Each VH, Vκ, and Vλ primer pair was added to a separate 50 µl reaction mix with an appropriate equimolar mixture of the four JH, two Jκ, or three Jλ reverse primers. Then, 0.5 ng cDNA was used in each PCR reaction using the Hot Start Taq Master Mix Kit (Evrogen, Russian Federation). The conditions of PCR were as follows: 1 step (94°C—3 min), 1 cycle (94°C—25 s, 62°C—25 s, 72°C—25 s), 2 cycles (94°C—25 s, 60°C—25 s, 72°C—25 s), 2 cycles (94°C—25 s, 58°C—25 s, 72°C—25 s), 3 cycles (94°C—25 s, 56°C—25 s, 72°C—25 s), 3 cycles (94°C—25 s, 54°C—25 s, 72°C—25 s), 30 cycles (94°C—25 s, 52°C—25 s, 72°C—25 s), and 1 step (72°C—4 min). The products of 15 PCR reactions for VH genes, 13 PCR reactions for Vκ genes, and 16 PCR reactions for Vλ genes were combined for each chain and concentrated to 50–80 µl using Amicon 30 kDa (Merck, Millipore). The PCR products (~400 bp length) of VH, Vκ, and Vλ were loaded on 1.5% agarose gels and purified with the Gel Extraction Kit (Monarch, NEB).

### Deep sequencing of VH and VL genes from individual patients

Next, 1 μg of the PCR product was ligated with adapters using the NEBNext Ultra DNA Library Prep Kit for Illumina with the NEBNext Multiplex Oligos set (NEB). Libraries were sequenced on Miseq using a 2x300 bp paired-ends sequencing kit (Illumina) in the SB RAS Genomics Core Facility (ICBFM SB RAS, Novosibirsk, Russia).

### Sequencing data processing and repertoire analysis

MiXCR (63) software was used to extract BCR clonotypes from raw sequencing data. Raw reads were aligned to the standard reference set of V, D (for heavy chain), and J gene-segment sequences. Successfully aligned reads were used for clonotype sequence assembly with the following parameters: OassemblingFeatures=“{CDR1Begin : CDR3End}” -OmaxBadPointsPercent=0. To normalize the repertoire analysis depth, equal numbers (13,000 for IGVH or IGVK and 7000 for IGVL) of read pairs covering the full target sequence (CDR1+FR2+CDR2+FR3+CDR3) were randomly sampled from each data set. A BCR clonotype is referred to here as a unique nucleotide sequence covering BCR from the beginning of CDR1 to the end of CDR3. Repertoire sequencing results are summarized in **Table S2**.

Repertoire data analysis was performed using the R programming language (R Core Team (2017) R: the language and environment for statistical computing (R Foundation for Statistical Computing, Vienna, Austria. URL https://www.R-project.org/). Unproductive IGVH/IGVK/IGVL sequences were filtered out before analysis. To characterize the germline identity for each clonotype, a nucleotide sequence covering the CDR1-FR3 part was used for calculating the percentage of identity with the corresponding reference V-segment sequence. The VDJ tools software (64) with the CalcCdrAaStats subroutine was used to obtain the statistics on composition and physical-chemical properties of amino acids in the CDR3 region. For CDR3 length analysis, we defined the length as a number of amino acids from conservative Cys at the end of the part encoded by the V-segment to the conservative Phe/Trp encoded by the J-segment (65).

### IL-10 secretion assay

B cells from isolated PBMC were enriched using magnetic Dynabeads (negative selection; Invitrogen, Thermo Fisher) following the manufacturer’s instructions with >95% purity. Enriched B cells were maintained in the complete glutamine-enriched RPMI-1640 medium supplemented with 10% fetal bovine serum (FBS) and 10 mM HEPES at a concentration of 0.5•10^6^ cells/mL in six-well culture plates at 37°C with 5% CO_2_. IL-10 production was induced by the incubation with 10 µg/mL CpG-B ODN 2006 for 20 hours. CpG-stimulated B cells were restimulated by adding up to 50 ng/mL PMA (Sigma-Aldrich) and 0.5 µg/mL ionomycin (Sigma-Aldrich) for four hours.

Stimulated B cells were washed twice with PBS and stained for assessing the cell viability with Zombie Violet Fixable Viability Kit (Biolegend, USA) according to the manufacturer’s instructions. Next, B cells were washed with cold (MACS) buffer containing PBS supplemented with 0.5% BSA and 2 mM EDTA; 10^6^ of B cells were then resuspended in 90 µl of cold RPMI-1640 medium supplemented with 10% FBS and incubated with 10 µl IL-10 Catch Reagent (IL-10 secretion assay, Miltenyi Biotec) for five minutes on ice. Subsequently, 1 mL of warm (37°C) RPMI-1640 medium supplemented with 10% FBS was added, and cells were kept for 45 minutes at 37°C under slow continuous rotation. B cells were washed twice with a cold (MACS) buffer, resuspended in 80 µl cold (MACS) buffer with the addition of 10 µl IL-10 detection antibody (APC) (IL-10 secretion assay, Miltenyi Biotec) and 10 µl of the antibody mix (α-CD19-PE-Cy7, α-CD24-PE, and α-CD38-AlexaFluor700). After 20 minutes of incubation on ice, B cells were washed with cold buffer and resuspended in PBS. IL-10^+^ and IL-10^−^ B cells were analyzed using FACS Aria III (BD Biosciences).

### Statistical analysis

The data were analyzed using Prism 9 software. The significance of differences was assessed using the two-tailed Student’s t-test, Mann Whitney U-test, or ANOVA. p-values <.05 were considered to be significant.

## Results

### The elevated level of transitional CD19^+^CD24^high^CD38^high^ Bregs in peripheral blood correlates with MS severity

**Figure 1:**
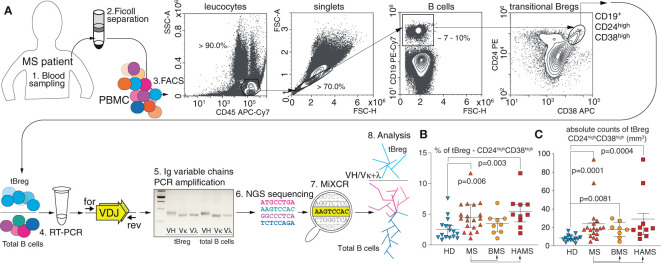
MS severity correlates with the elevated level of transitional CD19^+^CD24^high^CD38^high^ Bregs in peripheral blood. **(A)** Total CD19^+^ B cell pool and transitional regulatory CD19^+^CD24^high^CD38^high^ subpopulation (tBregs) from peripheral blood were sorted individually for subsequent RNA isolation and RT-PCR amplification of IGVH, IGVK and IGVL followed by high-throughput sequencing and subsequent bioinformatic clustering and analysis. **(B)** The percentage and **(C)** absolute cell count of transitional Bregs in MS patients (MS) and healthy donors (HD). BMS denotes benign multiple sclerosis, HAMS is highly active multiple sclerosis. Data are shown as mean values, interquartile range, and *p*-values. The statistical significance was evaluated with the Mann Whitney test (only significant *p*-values are shown).

We analyzed patients with two diverging courses of relapsing-remitting MS (RRMS): (*i*) BMS (57), characterized by an infrequent relapse and low levels of disability over long periods of time, and (*ii*) HAMS (58) with elevated levels of inflammatory activity and rapid progression that facilitate a shift to secondary progressive MS with severely disrupted control of the immune response. The peripheral blood samples were obtained from 19 MS patients and 16 HDs (**Table 1**). To gain further insight into the nature of Breg development and maturation, we analyzed the CD19^+^CD24^high^CD38^high^ subpopulation, which is the most studied phenotype of tBreg (26, 66). Mononuclear cells were stained against CD45, CD19, CD24, and CD38 markers. A gating strategy is shown in **Figure 1A**. We found that the cell number and frequency of CD19^+^CD24^high^CD38^high^ B cells were significantly increased in the MS patients (4.5 ± 2.4%) as compared with the HDs (2.6% ± 1.8%). This upregulation was most pronounced in the HAMS patients (5.5% ± 2.7%) (**Figures 1B, C**). Wherein we observed no differences in the absolute counts for B cells between HDs and MS patients with various courses of MS (**Table 2**).

**Table 2:** Total numbers and frequency of T1/T2 subpopulations of transitional B cells in peripheral blood from the MS patients and healthy donors.

Clinical group	HD	MS	BMS	HAMS
Absolute counts B cells per mm^3^	455 ± 52	508 ± 74	508 ± 98	508 ± 112
Absolute counts tBreg cells per mm^3^	8.4 ± 1.0*	24.0 ± 5.2*	17.7 ± 3.0*	29.1 ± 9.0*
T2/T1 ratio	2.8 ± 0.2	3.9 ± 0.7	3.2 ± 0.3	4.4 ± 1.1
Absolute counts T1 cells per mm^3^	2.3 ± 0.3*	6.6 ± 2.1*	3.5 ± 0.8	8.1 ± 3.1
Absolute counts T2 cells per mm^3^	6.1 ± 0.7*	16.3 ± 3.6*	9.9 ± 2.2	19.4 ± 5.1*

All values are expressed as mean values ± SEM. Significantly different values evaluated by a Mann Whitney test between the healthy donors and MS patients are indicated with asterisks.

Previously, Cherukuri and colleagues showed that the activity of regulatory B cells varies depending on the composition of the tBreg subpopulation (67). A reduced T2/T1 ratio is associated with elevated IL-10 production and the most efficient T cell suppression (61). We studied the ratio between the transitional T1 and T2 subsets (the gating strategy is shown on **Figure 4**) distinguished by CD24 and CD38 expression (**Table 2**). The absolute T1 count was significantly elevated in the MS patients (*p* = .037) in comparison with healthy donors. The absolute T2 counts in peripheral blood were also significantly increased in the MS patients as compared with HDs (*p* = .0007), especially during HAMS (*p* = .0002). Whereas the mean value of the T2/T1 ratio is elevated during MS progression, no statistically significant difference in comparison to healthy donors was observed. To carefully distinguish between T1 and T2 cells, we analyzed CD24 and CD38 expression as well as the expression of IgD, being an important marker of B cell developmental stage and maturation (68, 69). We observed no significant difference in the T2(CD24^high^CD38^high^IgD^+^)/T1(CD24^high^CD38^high^IgD^low/-^) ratio between the two groups of six MS and four HDs, respectively (**Figure S3**).

To characterize the BCR repertoire of tBregs cells, we sorted total CD19^+^ B cells and CD19^+^CD24^high^CD38^high^ subpopulations and performed high-throughput sequencing of BCR cDNA libraries for variable heavy (VH) and light (VL) chains in these cell subsets. On average, we obtained ~40,000 and ~37,000 IGVH- and IGVK-containing reads for tBregs and total B cells, respectively. We obtained ~25,000 and ~17,000 IGVL-containing reads for tBregs and total B cells, respectively. We achieved the minimum depth of two IG sequence-containing reads per cell in most samples. Heavy and light chain clonotypes were assembled by the MiXCR with sequencing error correction (63). To reduce a potential bias, we used the equal repertoire analysis depth for all individuals (see Material and Methods section). For the CD19^+^CD24^high^CD38^high^ B cell subpopulation, we obtained and included in our analysis ≈4500 functional clonotypes for the heavy chain, ≈3500 for the kappa, and ≈550 for lambda chains for individuals in the MS and HD cohorts. For total B cells (the CD19+ subpopulation), we obtained ≈4000 functional clonotypes for heavy chain, ≈4300 for kappa, and ≈830 for lambda chains from the raw sequencing data and included this in our analysis for the individuals from the MS and HD cohorts (**Table S2**).

### Transitional CD19^+^CD24^high^CD38^high^ Bregs of MS patients are characterized by a lower number of hypermutations compared with the healthy individuals

**Figure 2:**
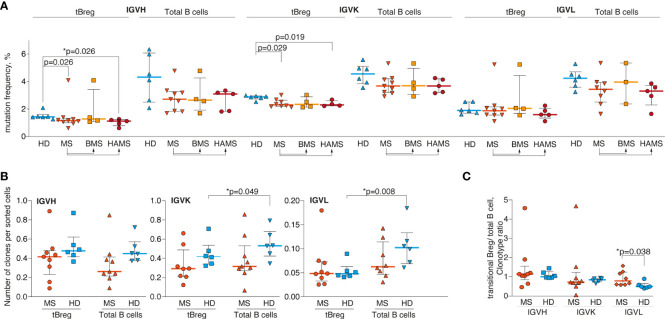
Delayed maturation of CD24^high^CD38^high^ transitional B lymphocytes in the MS patients. **(A)** Mutation frequency for V_H_, Vκ, and Vλ genes; **(B)** the number of unique clonotypes per sorted cells; and **(C)** the tBreg/total B cell clonotype ratio of the total blood B cells and CD24^high^CD38^high^ transitional Bregs from the multiple sclerosis patients (MS) and healthy donors (HD). Mutation frequency refers to the percentage of clonotype sequence different from the corresponding germline V- and J-segment sequences excluding CDR3 region. BMS denotes benign multiple sclerosis, HAMS is highly active multiple sclerosis. Bar and line plots represent a median and interquartile range. Statistical significance of the differences between donor groups was assessed using the Mann-Whitney test **(A, B)** and ratio paired *T*-test **(C)**. *p*-values <.05 after correction for multiple comparisons were considered statistically significant and designated with asterisks.

The tBregs are suggested to be the exogenous antigen-naïve cell population, therefore exhibiting fewer somatic hypermutations than the total pool of CD19^+^ B cells from peripheral blood (**Figure 2A**). V_H_ and Vκ genes of CD19^+^CD24^high^CD38^high^ tBregs from MS patients are generally less mutated as compared with the HDs. There was a statistically significant difference between the HDs and HAMS patients yet not between the donors and BMS patients (**Figure 2A**). These data are in line with an increased level of tBregs in the peripheral blood from the HAMS patients. We observed approximately the same number of the IGH clonotypes in tBregs and total CD19^+^ B cells in the MS patients and healthy donors (**Figure 2B**). Analysis of repertoire diversity of the light chains showed that in the HDs the ratio in number of the IGK and IGL clonotypes in the total CD19^+^ pool was higher than in tBregs, whereas it remained unchanged in MS patients (**Figure 2B**). The lowest repertoire diversity was observed for the lambda light chain. The ratio of tBreg/total B cell clonotypes between MS patients and HDs was different only for the lambda chain (**Figure 2C**).

Because in the MS patients we observed fewer somatic hypermutations in the tBreg clonotypes, one could suggest a higher number of CDR3 in this cell subset shared among MS patients. However, we detected only a few tBreg IGH clonotypes common for at least two different donors. Furthermore, we did not observe any significant differences in the number of shared amino acid CDR3 sequences between MS and HD (data not shown).

### Characteristics of the CDR3 region of tBreg clonotypes do not vary between MS and healthy states

CDR3 is the most variable region of immunoglobulin molecules. It can be used for identifying clonal lineages and characterizing their functional repertoire (70). First, we examined CDR3 characteristics by comparing the amino acid CDR3 length of the heavy, kappa, and lambda chains among HDs, HAMS, and BMS disease states in tBregs and total peripheral B cells. In line with our expectations, we detected a significant difference in amino acid length between the heavy chain (18.3 ± 0.7 a.a.) and both light chain types (11.2 ± 0.1 a.a. for the kappa chain and 11.5 ± 0.3 a.a. for the lambda chain) due to the presence of the D-segment in the IGH.

**Figure 3:**
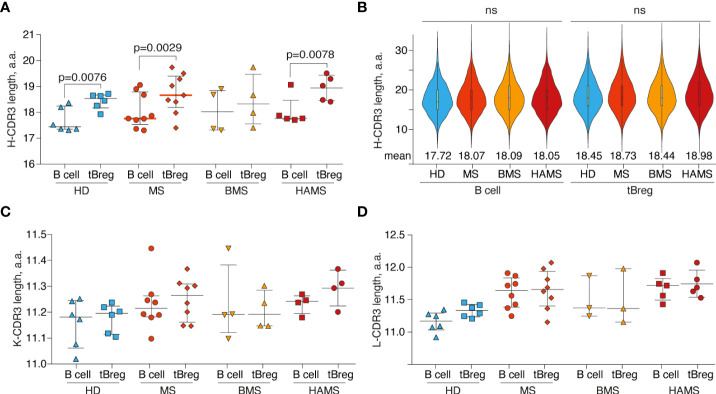
Differences in CDR3 length. The distribution of B cell subset CDR3 amino acid length for heavy **(A)**, kappa **(C)**, and lambda **(D)** light chains. Bar and line plots show mean ± SD. **(B)** The CDR3 amino acid length distribution for IGVH clonotypes in different B cell subsets. To balance the sample size, an equal number of clonotypes (*n* = 1000) were randomly sampled from each donor repertoire. Rare clonotypes with CDR3 length <6 a.a. or >35 a.a. were excluded. Mean values are displayed by numbers. The difference in CDR3 length between tBreg and total B cell fraction was analyzed by the ratio paired *T*-test. The difference in CDR3 length between donor groups was assessed using the Mann Whitney test. Only statistically significant *p*-values are indicated. The total CD19^+^ B cell pool and transitional regulatory CD19^+^CD24^high^CD38^high^ subpopulation from peripheral blood are designated as B cells and tBregs, respectively. ns , not significant.

For the heavy chain, the CDR3 length significantly varied between the tBreg and the total B cell populations (**Figure 3A**) in contrast to the previously reported data (71). Nonetheless, there was no statistically significant difference in the CDR3 length between the MS patients and healthy individuals (**Figure 3B**). We did not detect any significant differences in the length of CDR3 of the kappa and lambda light chains between the tBreg subset and total B cells (**Figures 3C, D**, **S1**).

Furthermore, we compared the physicochemical properties of the CDR3 amino acids for the clonotypes from tBregs and total peripheral B cells. We observed no significant differences in the charge and hydrophobicity or amino acid usage in the CDR3 regions between the B cell subsets of the MS patients and HDs (data not shown).

### The CD19^+^CD24^high^CD38^high^ subpopulation in MS patients is characterized by less mature phenotype

**Figure 4:**
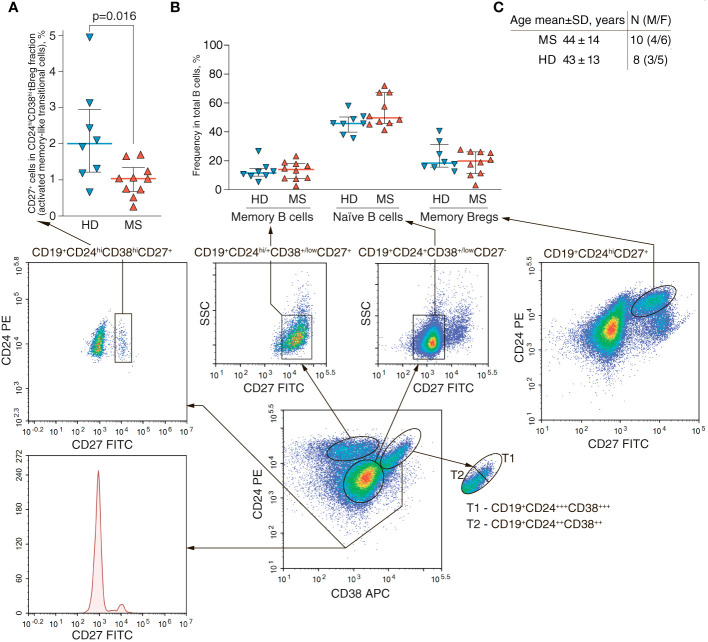
Frequencies of the CD27-positive B cells in peripheral blood of MS patients and healthy individuals. **(A)** The percentage of CD27-positive activated memory-like transitional cells in CD19^+^CD24^high^CD38^high^ tBreg subpopulation and **(B)** the frequency of memory (CD19^+^CD24^+/high^CD38^+/low^CD27^+^), naïve (CD19^+^CD24^+^CD38^+/low^CD27^-^) or memory Breg (CD24^high^CD27^+^) among peripheral B cells in multiple sclerosis patients (MS) and healthy donors (HD). The bottom panel shows the gating strategy of flow cytometric analysis. **(C)** Age and gender comparison of MS and HD analyzed in the same experiment. The difference in cell frequency was analyzed by Mann Whitney test. Statistically significant *p*-values are shown.

It is previously reported that the number of mature memory CD27^+^ peripheral B cells (72) in MS patients tends to decrease as compared with healthy individuals (73). Importantly, the ratio of different B cell populations and especially of the memory B cells varies with age (74). To avoid any age-related bias, the samples from sex- and age-matched MS patients (44 ± 14 y.o.) and HDs (43 ± 13 y.o.) were enrolled in this study (**Table 1**; **Figure 4C**). To examine the phenotypic stage of tBreg maturation, we analyzed the percentage of CD27 positive cells in this subpopulation (**Figure 4A**). We found that the CD27^+^ cell content in the CD19^+^CD24^high^CD38^high^ tBregs cells was significantly reduced in the MS patients (1.0% ± 0.5%) as compared with healthy individuals (2.2% ± 1.4%). We observed no correlation between age and the frequency of CD27^+^ cells in the CD19^+^CD24^high^CD38^high^ subpopulation (**Figure S2**). Our findings correlate well with previously reported data suggesting that Bregs (CD19^+^IL10^+^) mostly had the CD27^-^ phenotype in HD and MS patients in remission (28), whereas the percentage as well as the absolute counts of memory Bregs (CD24^high^CD27^+^) and naïve and memory B cells were similar in the HDs and MS patients (**Figure 4B**; **Table 3**).

**Table 3:** Total numbers of B cell subpopulations in peripheral blood of the MS patients and healthy donors (gating strategy is shown in **Figure 4**).

Clinical group	HD	MS	BMS	HAMS
Memory B cells	51 ± 9	41 ± 6	34 ± 5	46 ± 9
Naïve B cells	192 ± 40	216 ± 47	172 ± 42	246 ± 75
Memory Breg cells	89 ± 17	77 ± 22	44 ± 6	100 ± 34

All values are given as absolute counts per mm^3^ and presented as mean ± SEM.

### CD19^+^CD24^high^CD38^high^ B cells are characterized by an elevated production of IL-10 in MS patients and HDs

**Figure 5:**
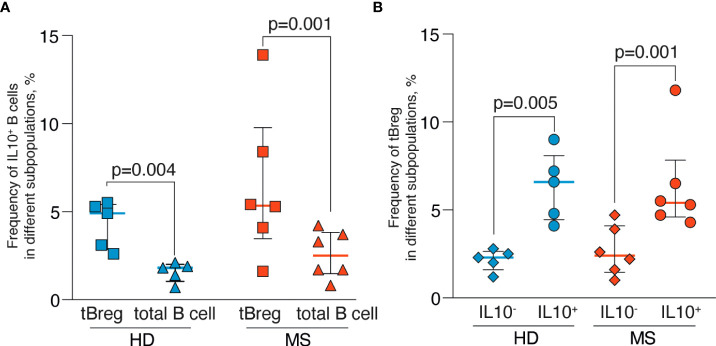
Frequencies of the IL-10–positive B cells in peripheral blood of MS patients and healthy individuals after rapid CpG stimulation. **(A)** The percentage of IL-10–positive cells in CD19^+^CD24^high^CD38^high^ tBreg subpopulation and total B cells. **(B)** The percentage of CD19^+^CD24^high^CD38^high^ tBreg in IL-10–positive and IL-10–negative B cells. Results are expressed as a median, interquartile range, and *p*-values analyzed by ratio paired *T*-test. Statistical significance of the differences between donor groups was assessed by the Mann Whitney test, and significance of differences between B cell subpopulations was assessed by the ratio paired *T*-test. Statistically significant *p*-values (*p* <.05) are shown.

Because the regulatory properties of B cells are not limited exclusively to the CD19^+^CD24^high^CD38^high^ subpopulation (55, 56), we analyzed the frequencies of the IL-10-positive cells among the total pool of B lymphocytes after a short CpG stimulation. Such rapid stimulation (less than 24 hours) allows estimating IL-10 production only in Bregs and not in the B cells predisposed for the IL-10 expression. There were no statistically significant differences in the IL-10–producing B cell subsets between MS and HD, whereas more IL-10–positive B cells were observed in the CD19^+^CD24^high^CD38^high^ subpopulation than in total B cells for both MS and HD (**Figure 5A**). Conversely, IL-10–positive B cells in MS and HD were enriched with CD19^+^CD24^high^CD38^high^ cells (**Figure 5B**).

## Discussion

The existence of several alternative methods of Breg differentiation might lead to different subsets of B cells with regulatory functions coexisting in an inflammatory milieu (75). The Breg population can originate from either multiple or a limited set of independent pre-Bregs progenitors. Furthermore, it results in either a diverse or restricted clonal repertoire, the latter being ensured by the clonally related Bregs. Moreover, almost any B cell can become a regulatory B cell at a certain stage of its development upon exposure to permissive environmental stimuli (35, 55). In the present study, we focus on the repertoire of tBregs with the CD19^+^CD24^high^CD38^high^ phenotype.

The mechanism of immune regulation mediated by B cells was first proposed by S. Fillatreau and colleagues. They first identified a subpopulation of B cells (B10 cells) that produced IL-10, alleviating the clinical manifestations of EAE (76). Bregs can also affect the differentiation of T cells to T regulatory cells (Tregs) (24) and inhibit differentiation of the effector T cells *via* the IL-10–driven suppression of dendritic cells (35). Bregs are shown to suppress inflammation by producing transforming growth factor-β (TGF-β) (77), IL-35 (i35 Breg) (78), IgM, IgG4, the co-inhibitory receptor TIGIT (T cell immunoreceptor with Ig and ITIM domains) (36), and *BTLA* (B and T lymphocyte attenuator or CD272) (79). Nonetheless, IL-10 production is still believed to be the key mechanism for Bregs to control the immune response in healthy individuals as well as during immune-related disorders (26, 27, 80–82) and organ transplantation (83).

Breg maturation may be induced *via* BCR and/or TLR signaling (84). Using EAE, the experimental mouse model of autoimmune diseases, a significant impact of TLR-signaling on B cells was revealed (85). TLR-4 and TLR-9 induce a significant increase in IL-10 secretion by B cells in a MyD88-dependent manner. Their activation *via* TLR and CD40 ligands is also described for infectious diseases of viral (HIV (31), HBV (86)), bacterial (87, 88), and parasitic origins (89). Exposure of PBMC from both healthy donors and patients with rheumatoid arthritis to CD40L and CpG led to an increased number of IL-10–producing Bregs (90). A significantly lower level of IL-10 production by B cells stimulated in the presence of CD40L was found in the groups with relapsing-remitting (29) and secondary-progressive MS as compared with HDs. A similar effect was observed in the CpG-stimulated B cells (82). The fact that CD40 ligation on B cells plays an important role in this process promoting cell survival (91) is in line with the inductive model of Breg formation. The latter implies that B cells may become regulatory and exhibit suppressive capacity in response to specific environmental stimuli.

Another mechanism to induce Bregs is to activate them through BCR signaling (76). Matsumoto et al. demonstrate the importance of correct antigen recognition by Breg BCR (92). The antigen-mediated calcium influx in Bregs is shown to depend on STIM-1 (stromal interaction molecule) and STIM-2. A deficiency in these molecules impairs the ability of Bregs to produce IL-10, disrupts T cell activation, and eventually prevents alleviating EAE in mice. The number of Bregs was reduced in CD19-deficient mice with disrupted BCR-signaling, whereas CD19 overexpression resulted in an increased number of B10 cells (93–95). The antigen-specific interaction between CD4^+^ T cells and B10 cells is crucial for generating B10 effector cells, whereas antigen-specific T cell response is downregulated by B10-mediated IL-10 production (96). Therefore, the B10 cell effector function primarily depends on antigen specificity; however, it might also be mediated by suppressing antigen presentation by dendritic cells and macrophages (97).

We observed that CD19^+^CD24^high^CD38^high^ tBregs have a significantly longer CDR3 in the heavy chain as compared with the total pool of peripheral B cells. Antigen-experienced B cells were previously shown to have shorter CDR3 than naïve or immature B cells (98, 99), thus indicating that, in tBregs, longer CDR3 can point at their immature state. This can also be related to the differences in V/J-usage between tBregs and total B cells (100, 101). Finally, in tBregs, longer CDR3 may reflect the higher poly-/self-reactive potential of their BCRs (102).

Another piece of evidence underlying the importance of BCR during Breg activation is that human B10 cells are often defined as CD27^+^. This allows for classifying them as memory cells, which is in line with their *in vivo* antigen experience (25)*.* Of note, in the RRMS patients at the relapse stage, the ratio of naïve and memory Bregs is decreased, resulting in an elevated memory/naïve ratio (28). The presence of the CD27^+^ subpopulation in early immature transitional B cells could be explained by the recently proposed class-switching of antibodies in early human B cell development (71). Therefore, even transitional cells can undergo early maturation or, according to the alternative hypothesis, the CD19^+^CD24^high^CD38^high^CD27^+^ subset might belong in the IgM memory population (103, 104). Here, we show that the CD19^+^CD24^high^CD38^high^ tBregs from MS patients contain a statistically lower number of CD27-positive cells as compared with HDs.

**Figure 6:**
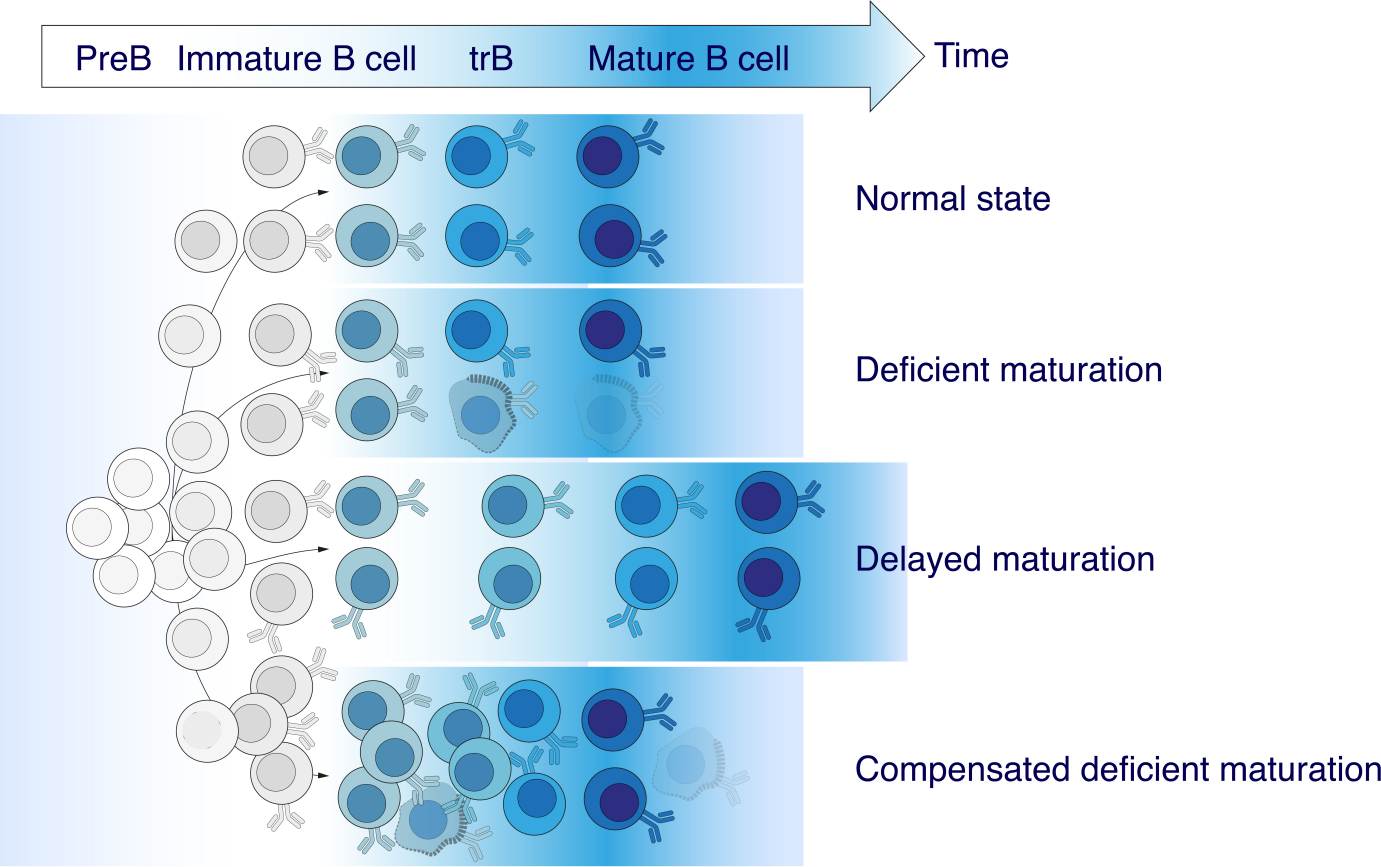
Abnormalities in transitional B cell maturation in MS. An elevated frequency of CD19^+^CD24^high^CD38^high^ transitional B cells (TrB) observed in MS patients is characterized by greater germline identity compared with healthy donors. There are least three possible coexisting or independent scenarios explaining these findings: (*i*) deficient maturation of TrB, (*ii*) delayed TrB maturation, and (*iii*) an increased number of TrB compensating deficient maturation.

Our findings allow us to conclude that the elevated absolute number and frequency of CD19^+^CD24^high^CD38^high^ tBregs observed in MS patients is characterized by a greater germline identity as compared with HD. At the same time, the absolute cell counts of the recently immigrated from the bone marrow T1 and more mature T2 cells both increased, thus more or less maintaining the overall T1/T2 ratio as in a healthy state. We propose at least three scenarios explaining these findings: (i) deficient maturation of transitional B cells (TrB); (ii) delayed TrB maturation, and (iii) elevated TrB counts that compensate for deficient maturation (**Figure 6**). The latter seems to be the most likely scenario because the T1/T2 ratio and, especially, percentage and absolute counts of TrB are increased in MS patients, whereas the numbers of memory and naïve B cells remain unaltered. Nonetheless, current evidence is not conclusive yet.

The tBregs maturation failure could arise from either an intrinsic defect in pre-Bregs or insufficient antigen-induced maturation. The BCR sequences of B10 cells were recently shown to be closer to germline and harbor only rare mutations (105). The authors state that, similarly to the splenic B10 cells, the peritoneal cavity B10 cells expressed clonally diverse BCRs that were predominantly germline encoded. Despite the germline proximity, B10 cells are shown to produce IgG as well as IgM (106). Thus, the so-termed “low differentiated” BCR may provide low-affinity antigen-BCR stimulation during chronic disease and development of the B10 precursor to B10, whereas a strong stimulation may switch the Breg precursor to another differentiation pathway (25, 94, 105). In the present work, we revealed no differences in IL-10 production between MS and HD *via* functional analysis. Therefore, increased tBreg counts along with the absence of increased IL-10 production can serve as indirect evidence in favor of their disrupted functioning and altered inflammatory profile during MS development. We suggest that, in future studies, tBregs should be analyzed for IL-10 production as well as co-expression of other cytokines as IL-10^+^ B cells are previously shown to co-express pro-inflammatory cytokines such as IL-6 and tumor necrosis factor alpha (TNFa) (55).

Here, we put forward that the observed stagnation in the maturation of the transitional Bregs potentially may be the first step in the fatal sequence of events leading to the systemic failure of humoral regulative immunity. Being accompanied by one or multiple factors, such as HLA haplotype, cytokine background, abnormal T cell negative selection, enhanced blood–brain barrier permeability, or lack of vitamin D, it may trigger the uncontrolled breakdown of the immunological tolerance toward myelin antigens. On the other hand, the elevated absolute count of tBregs and T1/T2 B cell subset ratio may be a sign of the already activated compensatory mechanisms, which may be monitored on the border of a clinically isolated syndrome and clinically defined multiple sclerosis. Further studies should elucidate if modulating the immunological checkpoints regulating B cell development may be regarded as opening up the avenue for putative therapeutical applications in MS treatment.

## Data availability statement

The datasets presented in this study can be found in online repositories. The names of the repository/repositories and accession number(s) can be found below: https://www.ebi.ac.uk/arrayexpress/, E-MTAB-10859.

## Ethics statement

The studies involving human participants were reviewed and approved by Local Ethic Committee of the Research Center of Neurology, Moscow, Russia. The patients/participants provided their written informed consent to participate in this study.

## Author contributions

YL, ABJr and AG designed the research and wrote the paper. MNZ, MI and TS performed blood sample acquisition, patient data management and MS diagnosis. YL, LO, MK, AT, MYZ and DS performed research. YL, IZ, LO, AM, ABJr and AG analyzed data. YR, IZ, DC, NB, VM, AF and SI made intellectual contributions to data analysis. All authors contributed to the article and approved the submitted version.

## Funding

This study was supported by Russian Science Foundation, grant #17-74-30019. Cell sorting experiments were carried out using the equipment provided by the IBCH core facility (CKP IBCH, supported by grant of the Ministry of Science and Higher Education of the Russian Federation no. 075-15-2020-807 (to DMC, in part of BCR repertoire analysis). ABJr would like to acknowledge personal fellowship MD-5902.2021.1.4 and Project 075-15-2021-1033 (13.2251.21.0111) for NGS.

## Conflict of interest

The authors declare that the research was conducted in the absence of any commercial or financial relationships that could be construed as a potential conflict of interest.

## Publisher’s note

All claims expressed in this article are solely those of the authors and do not necessarily represent those of their affiliated organizations, or those of the publisher, the editors and the reviewers. Any product that may be evaluated in this article, or claim that may be made by its manufacturer, is not guaranteed or endorsed by the publisher.

## References

[1] McGinley MP Goldschmidt CH Rae-Grant AD. Diagnosis and treatment of multiple sclerosis. JAMA (2021) 325:765. doi: 10.1001/jama.2020.26858 33620411

[2] Aharoni R Eilam R Schottlender N Radomir L Leistner-Segal S Feferman T. Glatiramer acetate increases T- and b -regulatory cells and decreases granulocyte-macrophage colony-stimulating factor (GM-CSF) in an animal model of multiple sclerosis. J Neuroimmunol (2020) 345:577281. doi: 10.1016/j.jneuroim.2020.577281 32534388

[3] From R Eilam R Bar-Lev DD Levin-Zaidman S Tsoory M LoPresti P. Oligodendrogenesis and myelinogenesis during postnatal development effect of glatiramer acetate. Glia (2014) 62:649–65. doi: 10.1002/glia.22632 24481644

[4] Belogurov A Kuzina E Kudriaeva A Kononikhin A Kovalchuk S Surina Y. Ubiquitin-independent proteosomal degradation of myelin basic protein contributes to development of neurodegenerative autoimmunity. FASEB J (2015) 29:1901–13. doi: 10.1096/fj.14-259333 PMC4415016 25634956

[5] Belogurov AA Stepanov AV Smirnov IV Melamed D Bacon A Mamedov AE. Liposome-encapsulated peptides protect against experimental allergic encephalitis. FASEB J (2013) 27:222–31. doi: 10.1096/fj.12-213975 PMC3528315 23047895

[6] Belogurov A Zakharov K Lomakin Y Surkov K Avtushenko S Kruglyakov P. CD206-targeted liposomal myelin basic protein peptides in patients with multiple sclerosis resistant to first-line disease-modifying therapies: A first-in-Human, proof-of-Concept dose-escalation study. Neurotherapeutics (2016) 13:895–904. doi: 10.1007/s13311-016-0448-0 27324388 PMC5081122

[7] Walo-Delgado PE Monreal E Medina S Quintana E de la Maza SS Fernández-Velasco JI. Role of b cell profile for predicting secondary autoimmunity in patients treated with alemtuzumab. Front Immunol (2021) 12:760546. doi: 10.3389/fimmu.2021.760546 34691084 PMC8531491

[8] Fernández-Velasco JI Kuhle J Monreal E Meca-Lallana V Meca-Lallana J Izquierdo G. Effect of ocrelizumab in blood leukocytes of patients with primary progressive MS. Neurol - Neuroimmunol Neuroinflamm (2021) 8:e940. doi: 10.1212/NXI.0000000000000940 33408167 PMC7862094

[9] Hohlfeld R Wekerle H. Immunological update on multiple sclerosis. Curr Opin Neurol (2001) 14:299–304. doi: 10.1097/00019052-200106000-00006 11371751

[10] Junker A Ivanidze J Malotka J Eiglmeier I Lassmann H Wekerle H. Multiple sclerosis: T-cell receptor expression in distinct brain regions. Brain (2007) 130:2789–99. doi: 10.1093/brain/awm214 17890278

[11] Hasler P Zouali M. B cell receptor signaling and autoimmunity. FASEB J (2001) 15:2085–98. doi: 10.1096/fj.00-0860rev 11641235

[12] Lomakin YA Zakharova MY Stepanov AV Dronina MA Smirnov IV Bobik TV. Heavy-light chain interrelations of MS-associated immunoglobulins probed by deep sequencing and rational variation. Mol Immunol (2014) 62:305–14. doi: 10.1016/j.molimm.2014.01.013 24534716

[13] Ponomarenko NA Durova OM Vorobiev II Belogurov AA Kurkova IN Petrenko AG. Autoantibodies to myelin basic protein catalyze site-specific degradation of their antigen. Proc Natl Acad Sci U.S.A. (2006) 103:281–6. doi: 10.1073/pnas.0509849103 PMC1324791 16387849

[14] Villar LM Sádaba MC Roldán E Masjuan J González-Porqué P Villarrubia N. Intrathecal synthesis of oligoclonal IgM against myelin lipids predicts an aggressive disease course in MS. J Clin Invest (2005) 115:187–94. doi: 10.1172/JCI22833 PMC539201 15630459

[15] Reali C Magliozzi R Roncaroli F Nicholas R Howell OW Reynolds R. B cell rich meningeal inflammation associates with increased spinal cord pathology in multiple sclerosis. Brain Pathol (2020) 30:779–93. doi: 10.1111/bpa.12841 PMC8018043 32243032

[16] Cencioni MT Mattoscio M Magliozzi R Bar-Or A Muraro PA. B cells in multiple sclerosis — from targeted depletion to immune reconstitution therapies. Nat Rev Neurol (2021) 17:399–414. doi: 10.1038/s41582-021-00498-5 34075251

[17] Mauri C Menon M. Human regulatory b cells in health and disease: Therapeutic potential. J Clin Invest (2017) 127:772–9. doi: 10.1172/JCI85113 PMC5330739 28248202

[18] Sokolov AV Shmidt AA Lomakin YA. B Cell regulation in autoimmune diseases. Acta Naturae (2018) 10:11–22. doi: 10.32607/20758251-2018-10-3-11-22 30397522 PMC6209408

[19] Katz SI Parker D Turk JL. B-cell suppression of delayed hypersensitivity reactions. Nature (1974) 251:550–1. doi: 10.1038/251550a0 4547522

[20] Wolf SD Dittel BN Hardardottir F Janeway CA. Experimental autoimmune encephalomyelitis induction in genetically b cell–deficient mice. J Exp Med (1996) 184:2271–8. doi: 10.1084/jem.184.6.2271 PMC2196394 8976182

[21] Matsushita T Horikawa M Iwata Y Tedder TF. Regulatory b cells (B10 cells) and regulatory T cells have independent roles in controlling experimental autoimmune encephalomyelitis initiation and late-phase immunopathogenesis. J Immunol (2010) 185:2240–52. doi: 10.4049/jimmunol.1001307 PMC3717968 20624940

[22] Mauri C Gray D Mushtaq N Londei M. Prevention of arthritis by interleukin 10–producing b cells. J Exp Med (2003) 197:489–501. doi: 10.1084/jem.20021293 12591906 PMC2193864

[23] Mangan NE van Rooijen N McKenzie ANJ Fallon PG. Helminth-modified pulmonary immune response protects mice from allergen-induced airway hyperresponsiveness. J Immunol (2006) 176:138–47. doi: 10.4049/jimmunol.176.1.138 16365404

[24] Flores-Borja F Bosma A Ng D Reddy V Ehrenstein MR Isenberg DA. CD19+CD24hiCD38hi b cells maintain regulatory T cells while limiting TH1 and TH17 differentiation. Sci Transl Med (2013) 5(173):173ra23. doi: 10.1126/scitranslmed.3005407 23427243

[25] Iwata Y Matsushita T Horikawa M DiLillo DJ Yanaba K Venturi GM. Characterization of a rare IL-10–competent b-cell subset in humans that parallels mouse regulatory B10 cells. Blood (2011) 117:530–41. doi: 10.1182/blood-2010-07-294249 PMC3031478 20962324

[26] Blair PA Noreña LY Flores-Borja F Rawlings DJ Isenberg DA Ehrenstein MR. CD19+CD24hiCD38hi b cells exhibit regulatory capacity in healthy individuals but are functionally impaired in systemic lupus erythematosus patients. Immunity (2010) 32:129–40. doi: 10.1016/j.immuni.2009.11.009 20079667

[27] Sun F Ladha SS Yang L Liu Q Shi SX Su N. Interleukin-10 producing-b cells and their association with responsiveness to rituximab in myasthenia gravis. Muscle Nerve (2014) 49:487–94. doi: 10.1002/mus.23951 23868194

[28] Knippenberg S Peelen E Smolders J Thewissen M Menheere P Cohen Tervaert JW. Reduction in IL-10 producing b cells (Breg) in multiple sclerosis is accompanied by a reduced naïve/memory breg ratio during a relapse but not in remission. J Neuroimmunol (2011) 239:80–6. doi: 10.1016/j.jneuroim.2011.08.019 21940055

[29] Cencioni MT Ali R Nicholas R Muraro PA. Defective CD19+CD24 hi CD38 hi transitional b-cell function in patients with relapsing-remitting MS. Mult Scler J (2021) 27:1187–97. doi: 10.1177/1352458520951536 32924828

[30] Ma S Satitsuksanoa P Jansen K Cevhertas L van de Veen W Akdis M. B regulatory cells in allergy. Immunol Rev (2021) 299:10–30. doi: 10.1111/imr.12937 33345311

[31] Siewe B Stapleton JT Martinson J Keshavarzian A Kazmi N Demarais PM. Regulatory b cell frequency correlates with markers of HIV disease progression and attenuates anti-HIV CD8 + T cell function *in vitro* . J Leukoc Biol (2013) 93:811–8. doi: 10.1189/jlb.0912436 PMC3629440 23434518

[32] Rosser EC Mauri C. Regulatory b cells: Origin, phenotype, and function. Immunity (2015) 42:607–12. doi: 10.1016/j.immuni.2015.04.005 25902480

[33] Hasan MM Thompson-Snipes L Klintmalm G Demetris AJ O’Leary J Oh S. CD24 hi CD38 hi and CD24 hi CD27 + human regulatory b cells display common and distinct functional characteristics. J Immunol (2019) 203:2110–20. doi: 10.4049/jimmunol.1900488 31511354

[34] van de Veen W Stanic B Yaman G Wawrzyniak M Söllner S Akdis DG. IgG4 production is confined to human IL-10–producing regulatory b cells that suppress antigen-specific immune responses. J Allergy Clin Immunol (2013) 131:1204–12. doi: 10.1016/j.jaci.2013.01.014 23453135

[35] Matsumoto M Baba A Yokota T Nishikawa H Ohkawa Y Kayama H. Interleukin-10-Producing plasmablasts exert regulatory function in autoimmune inflammation. Immunity (2014) 41:1040–51. doi: 10.1016/j.immuni.2014.10.016 25484301

[36] Hasan MM Nair SS O’Leary JG Thompson-Snipes L Nyarige V Wang J. Implication of TIGIT+ human memory b cells in immune regulation. Nat Commun (2021) 12:1534. doi: 10.1038/s41467-021-21413-y 33750787 PMC7943800

[37] Noh J Choi WS Noh G Lee JH. Presence of Foxp3-expressing CD19(+)CD5(+) b cells in human peripheral blood mononuclear cells: Human CD19(+)CD5(+)Foxp3(+) regulatory b cell (Breg). Immune Netw (2010) 10:247–9. doi: 10.4110/in.2010.10.6.247 PMC3026945 21286386

[38] Lindner S Dahlke K Sontheimer K Hagn M Kaltenmeier C Barth TFE. Interleukin 21–induced granzyme b–expressing b cells infiltrate tumors and regulate T cells. Cancer Res (2013) 73:2468–79. doi: 10.1158/0008-5472.CAN-12-3450 23384943

[39] Marie-Cardine A Divay F Dutot I Green A Perdrix A Boyer O. Transitional b cells in humans: Characterization and insight from b lymphocyte reconstitution after hematopoietic stem cell transplantation. Clin Immunol (2008) 127:14–25. doi: 10.1016/j.clim.2007.11.013 18191619

[40] Simon Q Pers J-O Cornec D Le Pottier L Mageed RA Hillion S. In-depth characterization of CD24 high CD38 high transitional human b cells reveals different regulatory profiles. J Allergy Clin Immunol (2016) 137:1577–1584.e10. doi: 10.1016/j.jaci.2015.09.014 26525227

[41] Guerrier T Labalette M Launay D Lee-Chang C Outteryck O Lefèvre G. Proinflammatory b-cell profile in the early phases of MS predicts an active disease. Neurol - Neuroimmunol Neuroinflamm (2018) 5:e431. doi: 10.1212/NXI.0000000000000431 29296635 PMC5745361

[42] von Büdingen H-C Palanichamy A Lehmann-Horn K Michel BA Zamvil SS. Update on the autoimmune pathology of multiple sclerosis: B-cells as disease-drivers and therapeutic targets. Eur Neurol (2015) 73:238–46. doi: 10.1159/000377675 PMC4401636 25824054

[43] McFarland HF. The b cell — old player, new position on the team. N Engl J Med (2008) 358:664–5. doi: 10.1056/NEJMp0708143 18272890

[44] Belogurov AA Kurkova IN Friboulet A Thomas D Misikov VK Zakharova MY. Recognition and degradation of myelin basic protein peptides by serum autoantibodies: Novel biomarker for multiple sclerosis. J Immunol (2008) 180:1258–67. doi: 10.4049/jimmunol.180.2.1258 18178866

[45] Lomakin Y Kudriaeva A Kostin N Terekhov S Kaminskaya A Chernov A. Diagnostics of autoimmune neurodegeneration using fluorescent probing. Sci Rep (2018) 8:12679. doi: 10.1038/s41598-018-30938-0 30139963 PMC6107501

[46] Gabibov AG Belogurov AA Lomakin YA Zakharova MY Avakyan ME Dubrovskaya VV. Combinatorial antibody library from multiple sclerosis patients reveals antibodies that cross-react with myelin basic protein and EBV antigen. FASEB J (2011) 25:4211–21. doi: 10.1096/fj.11-190769 21859892

[47] Lomakin Y Arapidi GP Chernov A Ziganshin R Tcyganov E Lyadova I. Exposure to the Epstein–Barr viral antigen latent membrane protein 1 induces myelin-reactive antibodies *in vivo* . Front Immunol (2017) 8:777. doi: 10.3389/fimmu.2017.00777 28729867 PMC5498468

[48] Tejada-Simon MV Zang YCQ Hong J Rivera VM Zhang JZ. Cross-reactivity with myelin basic protein and human herpesvirus-6 in multiple sclerosis. Ann Neurol (2003) 53:189–97. doi: 10.1002/ana.10425 12557285

[49] Tengvall K Huang J Hellström C Kammer P Biström M Ayoglu B. Molecular mimicry between anoctamin 2 and Epstein-Barr virus nuclear antigen 1 associates with multiple sclerosis risk. Proc Natl Acad Sci (2019) 116:16955–60. doi: 10.1073/pnas.1902623116 PMC6708327 31375628

[50] Bjornevik K Cortese M Healy BC Kuhle J Mina MJ Leng Y. Longitudinal analysis reveals high prevalence of Epstein-Barr virus associated with multiple sclerosis. Science (2022) 375:296–301. doi: 10.1126/science.abj8222 35025605

[51] Guo S Chen Q Liang X Mu M He J Fang Q. Reduced peripheral blood regulatory b cell levels are not associated with the expanded disability status scale score in multiple sclerosis. J Int Med Res (2018) 46:3970–8. doi: 10.1177/0300060518783083 PMC6136025 30025488

[52] Michel L Chesneau M Manceau P Genty A Garcia A Salou M. Unaltered regulatory b-cell frequency and function in patients with multiple sclerosis. Clin Immunol (2014) 155:198–208. doi: 10.1016/j.clim.2014.09.011 25267439

[53] Jiusheng Deng JH. Blood b cell and regulatory subset content in multiple sclerosis patients. J Mult Scler (2015) 2(2):1000139. doi: 10.4172/2376-0389.1000139 PMC4484600 26137596

[54] de Andrés C Tejera-Alhambra M Alonso B Valor L Teijeiro R Ramos-Medina R. New regulatory CD19+CD25+ b-cell subset in clinically isolated syndrome and multiple sclerosis relapse. changes after glucocorticoids. J Neuroimmunol (2014) 270:37–44. doi: 10.1016/j.jneuroim.2014.02.003 24662004

[55] Glass MC Glass DR Oliveria J-P Mbiribindi B Esquivel CO Krams SM. Human IL-10-producing b cells have diverse states that are induced from multiple b cell subsets. Cell Rep (2022) 39:110728. doi: 10.1016/j.celrep.2022.110728 35443184 PMC9107325

[56] Lighaam LC Unger P-PA Vredevoogd DW Verhoeven D Vermeulen E Turksma AW. *In vitro*-induced human IL-10+ b cells do not show a subset-defining marker signature and plastically Co-express IL-10 with pro-inflammatory cytokines. Front Immunol (2018) 9:1913. doi: 10.3389/fimmu.2018.01913 30258433 PMC6143818

[57] Schaefer LM Poettgen J Fischer A Gold S Stellmann JP Heesen C. Impairment and restrictions in possibly benign multiple sclerosis. Brain Behav (2019) 9(4):e01259. doi: 10.1002/brb3.1259 30884218 PMC6456783

[58] Díaz C Zarco LA Rivera DM. Highly active multiple sclerosis: An update. Mult Scler Relat Disord (2019) 30:215–24. doi: 10.1016/j.msard.2019.01.039 30822617

[59] Kurtzke JF. Rating neurologic impairment in multiple sclerosis: An expanded disability status scale (EDSS). Neurology (1983) 33:1444–52. doi: 10.1212/WNL.33.11.1444 6685237

[60] Sanz I Wei C Jenks SA Cashman KS Tipton C Woodruff MC. Challenges and opportunities for consistent classification of human b cell and plasma cell populations. Front Immunol (2019) 10:2458. doi: 10.3389/fimmu.2019.02458 31681331 PMC6813733

[61] Burton H Dorling A. Transitional b cell subsets–a convincing predictive biomarker for allograft loss? Kidney Int (2017) 91:18–20. doi: 10.1016/j.kint.2016.10.028 28003081

[62] Cheng J Torkamani A Grover RK Jones TM Ruiz DI Schork NJ. Ectopic b-cell clusters that infiltrate transplanted human kidneys are clonal. Proc Natl Acad Sci (2011) 108:5560–5. doi: 10.1073/pnas.1101148108 PMC3078383 21415369

[63] Bolotin DA Poslavsky S Mitrophanov I Shugay M Mamedov IZ Putintseva EV. MiXCR: Software for comprehensive adaptive immunity profiling. Nat Methods (2015) 12:380–1. doi: 10.1038/nmeth.3364 25924071

[64] Shugay M Bagaev DV Turchaninova MA Bolotin DA Britanova OV Putintseva EV. VDJtools: Unifying post-analysis of T cell receptor repertoires. PloS Comput Biol (2015) 11:e1004503. doi: 10.1371/journal.pcbi.1004503 26606115 PMC4659587

[65] Lefranc M-P Pommié C Ruiz M Giudicelli V Foulquier E Truong L. IMGT unique numbering for immunoglobulin and T cell receptor variable domains and ig superfamily V-like domains. Dev Comp Immunol (2003) 27:55–77. doi: 10.1016/s0145-305x(02)00039-3 12477501

[66] Zhu H-Q Xu R-C Chen Y-Y Yuan H-J Cao H Zhao X-Q. Impaired function of CD19 + CD24 hi CD38 hi regulatory b cells in patients with pemphigus. Br J Dermatol (2015) 172:101–10. doi: 10.1111/bjd.13192 24935080

[67] Cherukuri A Salama AD Carter CR Landsittel D Arumugakani G Clark B. Reduced human transitional b cell T1/T2 ratio is associated with subsequent deterioration in renal allograft function. Kidney Int (2017) 91:183–95. doi: 10.1016/j.kint.2016.08.028 28029430

[68] Duong BH Ota T Aït-Azzouzene D Aoki-Ota M Vela JL Huber C. Peripheral b cell tolerance and function in transgenic mice expressing an IgD superantigen. J Immunol (2010) 184:4143–58. doi: 10.4049/jimmunol.0903564 PMC2874719 20231687

[69] Zhou Y Zhang Y Han J Yang M Zhu J Jin T. Transitional b cells involved in autoimmunity and their impact on neuroimmunological diseases. J Transl Med (2020) 18:131. doi: 10.1186/s12967-020-02289-w 32183811 PMC7079408

[70] Chaudhary N Wesemann DR. Analyzing immunoglobulin repertoires. Front Immunol (2018) 9:462. doi: 10.3389/fimmu.2018.00462 29593723 PMC5861150

[71] Mitsunaga EM Snyder MP. Deep characterization of the human antibody response to natural infection using longitudinal immune repertoire sequencing. Mol Cell Proteomics (2020) 19:278–93. doi: 10.1074/mcp.RA119.001633 PMC7000125 31767621

[72] Klein U Rajewsky K Küppers R. Human immunoglobulin (Ig)M+IgD+ peripheral blood b cells expressing the CD27 cell surface antigen carry somatically mutated variable region genes: CD27 as a general marker for somatically mutated (memory) b cells. J Exp Med (1998) 188:1679–89. doi: 10.1084/jem.188.9.1679 PMC2212515 9802980

[73] Niino M Hirotani M Miyazaki Y Sasaki H. Memory and naïve b-cell subsets in patients with multiple sclerosis. Neurosci Lett (2009) 464:74–8. doi: 10.1016/j.neulet.2009.08.010 19666086

[74] Ciocca M Zaffina S Fernandez Salinas A Bocci C Palomba P Conti MG. Evolution of human memory b cells from childhood to old age. Front Immunol (2021) 12:690534. doi: 10.3389/fimmu.2021.690534 34367150 PMC8343175

[75] Mauri C Menon M. The expanding family of regulatory b cells. Int Immunol (2015) 27:479–86. doi: 10.1093/intimm/dxv038 PMC4587489 26071023

[76] Fillatreau S Sweenie CH McGeachy MJ Gray D Anderton SM. B cells regulate autoimmunity by provision of IL-10. Nat Immunol (2002) 3:944–50. doi: 10.1038/ni833 12244307

[77] Natarajan P Singh A McNamara JT Secor ER Guernsey LA Thrall RS. Regulatory b cells from hilar lymph nodes of tolerant mice in a murine model of allergic airway disease are CD5+, express TGF-β, and co-localize with CD4+Foxp3+ T cells. Mucosal Immunol (2012) 5:691–701. doi: 10.1038/mi.2012.42 22718263 PMC3480990

[78] Zhang Y Li J Zhou N Zhang Y Wu M Xu J. The unknown aspect of BAFF: Inducing IL-35 production by a CD5+CD1dhiFcγRIIbhi regulatory b-cell subset in lupus. J Invest Dermatol (2017) 137:2532–43. doi: 10.1016/j.jid.2017.07.843 28844943

[79] Murphy KM Nelson CA Šedý JR. Balancing co-stimulation and inhibition with BTLA and HVEM. Nat Rev Immunol (2006) 6:671–81. doi: 10.1038/nri1917 16932752

[80] Oleinika K Mauri C Salama AD. Effector and regulatory b cells in immune-mediated kidney disease. Nat Rev Nephrol (2019) 15:11–26. doi: 10.1038/s41581-018-0074-7 30443016

[81] Duddy M Niino M Adatia F Hebert S Freedman M Atkins H. Distinct effector cytokine profiles of memory and naive human b cell subsets and implication in multiple sclerosis. J Immunol (2007) 178:6092–9. doi: 10.4049/jimmunol.178.10.6092 17475834

[82] Hirotani M Niino M Fukazawa T Kikuchi S Yabe I Hamada S. Decreased IL-10 production mediated by toll-like receptor 9 in b cells in multiple sclerosis. J Neuroimmunol (2010) 221:95–100. doi: 10.1016/j.jneuroim.2010.02.012 20227772

[83] Luo Y Luo F Zhang K Wang S Zhang H Yang X. Elevated circulating IL-10 producing breg, but not regulatory b cell levels, restrain antibody-mediated rejection after kidney transplantation. Front Immunol (2020) 11:627496. doi: 10.3389/fimmu.2020.627496 33584730 PMC7877339

[84] Dai Y-C Zhong J Xu J-F. Regulatory b cells in infectious disease. Mol Med Rep (2017) 16:3–10. doi: 10.3892/mmr.2017.6605 28534949 PMC5482109

[85] Lampropoulou V Hoehlig K Roch T Neves P Gómez EC Sweenie CH. TLR-activated b cells suppress T cell-mediated autoimmunity. J Immunol (2008) 180:4763–73. doi: 10.4049/jimmunol.180.7.4763 18354200

[86] Das A Ellis G Pallant C Lopes AR Khanna P Peppa D. IL-10–producing regulatory b cells in the pathogenesis of chronic hepatitis b virus infection. J Immunol (2012) 189:3925–35. doi: 10.4049/jimmunol.1103139 PMC3480715 22972930

[87] Horikawa M Weimer ET DiLillo DJ Venturi GM Spolski R Leonard WJ. Regulatory b cell (B10 cell) expansion during listeria infection governs innate and cellular immune responses in mice. J Immunol (2013) 190:1158–68. doi: 10.4049/jimmunol.1201427 PMC3552111 23275601

[88] Neves P Lampropoulou V Calderon-Gomez E Roch T Stervbo U Shen P. Signaling *via* the MyD88 adaptor protein in b cells suppresses protective immunity during salmonella typhimurium infection. Immunity (2010) 33:777–90. doi: 10.1016/j.immuni.2010.10.016 21093317

[89] Hussaarts L van der Vlugt LEPM Yazdanbakhsh M Smits HH. Regulatory b-cell induction by helminths: Implications for allergic disease. J Allergy Clin Immunol (2011) 128:733–9. doi: 10.1016/j.jaci.2011.05.012 21684587

[90] Bankó Z Pozsgay J Szili D Tóth M Gáti T Nagy G. Induction and differentiation of IL-10–producing regulatory b cells from healthy blood donors and rheumatoid arthritis patients. J Immunol (2017) 198:1512–20. doi: 10.4049/jimmunol.1600218 28087671

[91] Tsubata T Wu J Honjo T. B-cell apoptosis induced by antigen receptor crosslinking is blocked by a T-cell signal through CD40. Nature (1993) 364:645–8. doi: 10.1038/364645a0 7688865

[92] Matsumoto M Fujii Y Baba A Hikida M Kurosaki T Baba Y. The calcium sensors STIM1 and STIM2 control b cell regulatory function through interleukin-10 production. Immunity (2011) 34:703–14. doi: 10.1016/j.immuni.2011.03.016 21530328

[93] Yanaba K Bouaziz J-D Haas KM Poe JC Fujimoto M Tedder TF. A regulatory b cell subset with a unique CD1dhiCD5+ phenotype controls T cell-dependent inflammatory responses. Immunity (2008) 28:639–50. doi: 10.1016/j.immuni.2008.03.017 18482568

[94] Yanaba K Bouaziz J-D Matsushita T Tsubata T Tedder TF. The development and function of regulatory b cells expressing IL-10 (B10 cells) requires antigen receptor diversity and TLR signals. J Immunol (2009) 182:7459–72. doi: 10.4049/jimmunol.0900270 PMC3733128 19494269

[95] Tedder TF. B10 cells: A functionally defined regulatory b cell subset. J Immunol (2015) 194:1395–401. doi: 10.4049/jimmunol.1401329 25663677

[96] Lykken JM Candando KM Tedder TF. Regulatory B10 cell development and function: Fig. L Int Immunol (2015) 27:471–7. doi: 10.1093/intimm/dxv046 PMC4817073 26254185

[97] Mittal SK Roche PA. Suppression of antigen presentation by IL-10. Curr Opin Immunol (2015) 34:22–7. doi: 10.1016/j.coi.2014.12.009 PMC4444374 25597442

[98] Kitaura K Yamashita H Ayabe H Shini T Matsutani T Suzuki R. Different somatic hypermutation levels among antibody subclasses disclosed by a new next-generation sequencing-based antibody repertoire analysis. Front Immunol (2017) 8:389. doi: 10.3389/fimmu.2017.00389 28515723 PMC5413556

[99] Galson JD Trück J Fowler A Clutterbuck EA Münz M Cerundolo V. Analysis of b cell repertoire dynamics following hepatitis b vaccination in humans, and enrichment of vaccine-specific antibody sequences. EBioMedicine (2015) 2:2070–9. doi: 10.1016/j.ebiom.2015.11.034 PMC4703725 26844287

[100] Kaplinsky J Li A Sun A Coffre M Koralov SB Arnaout R. Antibody repertoire deep sequencing reveals antigen-independent selection in maturing b cells. Proc Natl Acad Sci (2014) 111:E2622–9. doi: 10.1073/pnas.1403278111 PMC4078805 24927543

[101] Sankar K Hoi KH Hötzel I. Dynamics of heavy chain junctional length biases in antibody repertoires. Commun Biol (2020) 3:207. doi: 10.1038/s42003-020-0931-3 32358517 PMC7195405

[102] Meffre E Milili M Blanco-Betancourt C Antunes H Nussenzweig MC Schiff C. Immunoglobulin heavy chain expression shapes the b cell receptor repertoire in human b cell development. J Clin Invest (2001) 108:879–86. doi: 10.1172/JCI13051 PMC200933 11560957

[103] Boyd SD Liu Y Wang C Martin V Dunn-Walters DK. Human lymphocyte repertoires in ageing. Curr Opin Immunol (2013) 25:511–5. doi: 10.1016/j.coi.2013.07.007 PMC4811628 23992996

[104] Martin VG Wu Y-CB Townsend CL Lu GHC O’Hare JS Mozeika A. Transitional b cells in early human b cell development – time to revisit the paradigm? Front Immunol (2016) 7:546. doi: 10.3389/fimmu.2016.00546 27994589 PMC5133252

[105] Maseda D Candando KM Smith SH Kalampokis I Weaver CT Plevy SE. Peritoneal cavity regulatory b cells (B10 cells) modulate IFN-γ + CD4 + T cell numbers during colitis development in mice. J Immunol (2013) 191:2780–95. doi: 10.4049/jimmunol.1300649 PMC3770313 23918988

[106] Maseda D Smith SH DiLillo DJ Bryant JM Candando KM Weaver CT. Regulatory B10 cells differentiate into antibody-secreting cells after transient IL-10 production *in vivo* . J Immunol (2012) 188:1036–48. doi: 10.4049/jimmunol.1102500 PMC3262922 22198952

