# Supplementary material for: Deconvolution of B cell receptor repertoire in multiple sclerosis patients revealed a delay in tBreg maturation
Source: Front Immunol. 2022 Aug 16;13:803229. doi: 10.3389/fimmu.2022.803229 (PMC9425031; doi:10.3389/fimmu.2022.803229)
Supplement: Supplementary file 1 [file DataSheet_1.docx]

**Supporting information.**

**Deconvolution of B cell receptor repertoire in Multiple Sclerosis patients revealed а delay in tBreg maturation.**

**Table S1. List of primers for deep sequencing individual VH, VK and VL genes.**

| № | name | Nucleotide sequence |  |
| --- | --- | --- | --- |
| 1 | VH1aSal | TAAGGCCCAGCCGGCCCAGGTKCAGCTGGTGCAGTCTGG | for VH |
| 2 | VH1bSal | TAAGGCCCAGCCGGCCCAGGTCCAGCTTGTGCAGTCTGG | for VH |
| 3 | VH1cSal | TAAGGCCCAGCCGGCCSAGGTCCAGCTGGTACAGTCTGG | for VH |
| 4 | VH1dSal | TAAGGCCCAGCCGGCCCARATGCAGCTGGTGCAGTCTGG | for VH |
| 5 | VH2aSal | TAAGGCCCAGCCGGCCCAGATCACCTTGAAGGAGTCTGG | for VH |
| 6 | VH2bSal | TAAGGCCCAGCCGGCCCAGGTCACCTTGARGGAGTCTGG | for VH |
| 7 | VH3aSal | TAAGGCCCAGCCGGCCGARGTGCAGCTGGTGGAGTCTGG | for VH |
| 8 | VH3bSal | TAAGGCCCAGCCGGCCCAGGTGCAGCTGGTGGAGTCTGG | for VH |
| 9 | VH3cSal | TAAGGCCCAGCCGGCCGAGGTGCAGCTGTTGGAGTCTGG | for VH |
| 10 | VH3dSal | TAAGGCCCAGCCGGCCGAGGTGCAGCTGGTGGAGTCTGG | for VH |
| 11 | VH4aSal | TAAGGCCCAGCCGGCCCAGSTGCAGCTGCAGGAGTCSGG | for VH |
| 12 | VH4bSal | TAAGGCCCAGCCGGCCCAGGTGCAGCTACAGCAGTGGGG | for VH |
| 13 | VH5aSal | TAAGGCCCAGCCGGCCGARGTGCAGCTGGTGCAGTCTGG | for VH |
| 14 | VH6aSal | TAAGGCCCAGCCGGCCCAGGTACAGCTGCAGCAGTCAGG | for VH |
| 15 | VH7aSal | TAAGGCCCAGCCGGCCCAGGTSCAGCTGGTGCAATCTGG | for VH |
| 16 | JH1-2Bss | GCCACCGCCGCCGCCTGAGCTGAGGAGACRGTGACCAGGGTGCC | rev VH |
| 17 | JH3Bss | GCCACCGCCGCCGCCTGAGCTGARGAGACGGTGACCATTGTCCC | rev VH |
| 18 | JH4-5Bss | GCCACCGCCGCCGCCTGAGCTGAGGAGACGGTGACCAGGGTTCC | rev VH |
| 19 | JH6Bss | GCCACCGCCGCCGCCTGAGCTGAGGAGACGGTGACCGTGGTCCC | rev VH |
| 20 | Vk1aXba | TCAGGCGGCGGCGGTGGCRACATCCAGATGACCCAGTCTCC | for Vk |
| 21 | Vk1bXba | TCAGGCGGCGGCGGTGGCGMCATCCAGTTGACCCAGTCTCC | for Vk |
| 22 | Vk1cXba | TCAGGCGGCGGCGGTGGCGCCATCCRGATGACCCAGTCTCC | for Vk |
| 23 | Vk1dXba | TCAGGCGGCGGCGGTGGCGTCATCTGGATGACCCAGTCTCC | for Vk |
| 24 | Vk2aXba | TCAGGCGGCGGCGGTGGCGATATTGTGATGACCCAGACTCC | for Vk |
| 25 | Vk2bXba | TCAGGCGGCGGCGGTGGCGATRTTGTGATGACTCAGTCTCC | for Vk |
| 26 | Vk3aXba | TCAGGCGGCGGCGGTGGCGAAATTGTGTTGACRCAGTCTCC | for Vk |
| 27 | Vk3bXba | TCAGGCGGCGGCGGTGGCGAAATAGTGATGACGCAGTCTCC | for Vk |
| 28 | Vk3cXba | TCAGGCGGCGGCGGTGGCGAAATTGTAATGACACAGTCTCC | for Vk |
| 29 | Vk4aXba | TCAGGCGGCGGCGGTGGCGACATCGTGATGACCCAGTCTCC | for Vk |
| 30 | Vk5aXba | TCAGGCGGCGGCGGTGGCGAAACGACACTCACGCAGTCTCC | for Vk |
| 31 | Vk6aXba | TCAGGCGGCGGCGGTGGCGAAATTGTGCTGACTCAGTCTCC | for Vk |
| 32 | Vk6bXba | TCAGGCGGCGGCGGTGGCGATGTTGTGATGACACAGTCTCC | for Vk |
| 33 | VL1aXba | TCAGGCGGCGGCGGTGGCCAGTCTGTGCTGACTCAGCCRCC | for Vl |
| 34 | VL1bXba | TCAGGCGGCGGCGGTGGCCAGTCTGTGYTGACGCAGCCRCC | for Vl |
| 35 | VL1cXba | TCAGGCGGCGGCGGTGGCCAGTCTGTCGTGACGCAGCCRCC | for Vl |
| 36 | VL2Xba | TCAGGCGGCGGCGGTGGCCAGTCTGCCCTGACTCAGCCT | for Vl |
| 37 | VL3aXba | TCAGGCGGCGGCGGTGGCTCCTATGWGCTGACTCAGCCACC | for Vl |
| 38 | VL3bXba | TCAGGCGGCGGCGGTGGCTCCTATGAGCTGACACAGCYACC | for Vl |
| 39 | VL3cXba | TCAGGCGGCGGCGGTGGCTCTTCTGAGCTGACTCAGGACCC | for Vl |
| 40 | VL3dXba | TCAGGCGGCGGCGGTGGCTCCTATGAGCTGATGCAGCCACC | for Vl |
| 41 | VL4aXba | TCAGGCGGCGGCGGTGGCCAGCYTGTGCTGACTCAATCRYC | for Vl |
| 42 | VL4bXba | TCAGGCGGCGGCGGTGGCCTGCCTGTGCTGACTCAGCCCCC | for Vl |
| 43 | VL5Xba | TCAGGCGGCGGCGGTGGCCAGSCTGTGCTGACTCAGCCRBC | for Vl |
| 44 | VL6Xba | TCAGGCGGCGGCGGTGGCAATTTTATGCTGACTCAGCCCCAC | for Vl |
| 45 | VL7Xba | TCAGGCGGCGGCGGTGGCCAGRCTGTGGTGACTCAGGAGCC | for Vl |
| 46 | VL8Xba | TCAGGCGGCGGCGGTGGCCAGACTGTGGTGACCCAGGAGCC | for Vl |
| 47 | VL4/9Xba | TCAGGCGGCGGCGGTGGCCWGCCTGTGCTGACTCAGCCACC | for Vl |
| 48 | VL10Xba | TCAGGCGGCGGCGGTGGCCAGGCAGGGCTGACTCAGCCACC | for Vl |
| 49 | VK1-4Hind | GCCAGGCCCCCGAGGCCACGTTTGATHTCCASYTTGGTCCC | rev VK |
| 50 | VK5Hind | GCCAGGCCCCCGAGGCCACGTTTAATCTCCAGTCGTGTCCC | rev VK |
| 51 | VL1Hind | GCCAGGCCCCCGAGGCCACCTAGGACGGTGACCTTGGTCCC | Rev Vl |
| 52 | VL2-3Hind | GCCAGGCCCCCGAGGCCACCTAGGACGGTCAGCTTGGTCCC | Rev Vl |
| 53 | VL7Hind | GCCAGGCCCCCGAGGCCACCGAGGACGGTCAGCTGGGTCCC | Rev Vl |

**Table S2. Summarized deep sequencing data for individual samples.**

| **donor ID** | **MS phenotype** | **cell population** | **IG chain** | **Cells sorted** | **Raw reads containing IG sequence** | **Number of reads sampled** | **Number of clones after downsampling** |
| --- | --- | --- | --- | --- | --- | --- | --- |
| HD1 | HD | Breg | IGH | 8800 | 35539 | 13000 | 4088 |
| HD1 | HD | Breg | IGK | 8800 | 53668 | 13000 | 3736 |
| HD1 | HD | Breg | IGL | 8800 | 30541 | 7000 | 396 |
| HD1 | HD | total | IGH | 8800 | 30052 | 13000 | 3927 |
| HD1 | HD | total | IGK | 8800 | 39295 | 13000 | 4696 |
| HD1 | HD | total | IGL | 8800 | 10940 | 7000 | 1018 |
| HD2 | HD | Breg | IGH | 13000 | 63684 | 13000 | 7005 |
| HD2 | HD | Breg | IGK | 13000 | 36192 | 13000 | 4505 |
| HD2 | HD | Breg | IGL | 13000 | 10885 | 7000 | 703 |
| HD2 | HD | total | IGH | 13000 | 49089 | 13000 | 4860 |
| HD2 | HD | total | IGK | 13000 | 55549 | 13000 | 5859 |
| HD2 | HD | total | IGL | 13000 | 26882 | 7000 | 792 |
| HD3 | HD | Breg | IGH | 13000 | 40447 | 13000 | 5627 |
| HD3 | HD | Breg | IGK | 13000 | 51894 | 13000 | 4205 |
| HD3 | HD | Breg | IGL | 13000 | 27803 | 7000 | 536 |
| HD3 | HD | total | IGH | 13000 | 63319 | 13000 | 5878 |
| HD3 | HD | total | IGK | 13000 | 38616 | 13000 | 4513 |
| HD3 | HD | total | IGL | 13000 | 18217 | 7000 | 937 |
| HD4 | HD | Breg | IGH | 4900 | 46663 | 13000 | 4274 |
| HD4 | HD | Breg | IGK | 4900 | 95434 | 13000 | 3468 |
| HD4 | HD | Breg | IGL | 4900 | 63361 | 7000 | 439 |
| HD4 | HD | total | IGH | 4900 | 37692 | 13000 | 3533 |
| HD4 | HD | total | IGK | 4900 | 19829 | 13000 | 3884 |
| HD4 | HD | total | IGL | 4900 | 7936 | 7000 | 904 |
| HD5 | HD | Breg | IGH | 6700 | 48427 | 13000 | 2457 |
| HD5 | HD | Breg | IGK | 6700 | 54719 | 13000 | 2765 |
| HD5 | HD | Breg | IGL | 6700 | 36849 | 7000 | 292 |
| HD5 | HD | total | IGH | 6700 | 47760 | 13000 | 2548 |
| HD5 | HD | total | IGK | 6700 | 44400 | 13000 | 4306 |
| HD5 | HD | total | IGL | 6700 | 22143 | 7000 | 665 |
| HD6 | HD | Breg | IGH | 8900 | 48942 | 13000 | 4372 |
| HD6 | HD | Breg | IGK | 8900 | 28836 | 13000 | 4259 |
| HD6 | HD | Breg | IGL | 8900 | 26644 | 7000 | 456 |
| HD6 | HD | total | IGH | 8900 | 31386 | 13000 | 4658 |
| HD6 | HD | total | IGK | 8900 | 66452 | 13000 | 4713 |
| HD6 | HD | total | IGL | 8900 | 16428 | 7000 | 939 |
| MS1 | BMS | Breg | IGH | 15000 | 15575 | 13000 | 2308 |
| MS1 | BMS | Breg | IGK | 15000 | 21830 | 13000 | 3266 |
| MS1 | BMS | Breg | IGL | 15000 | 9868 | 7000 | 402 |
| MS1 | BMS | total | IGH | 15000 | 42473 | 13000 | 3627 |
| MS1 | BMS | total | IGK | 15000 | 23708 | 13000 | 4558 |
| MS1 | BMS | total | IGL | 15000 | 4253 | 7000 | NA* |
| MS2 | BMS | Breg | IGH | 7000 | 21807 | 13000 | 2195 |
| MS2 | BMS | Breg | IGK | 7000 | 34074 | 13000 | 2061 |
| MS2 | BMS | Breg | IGL | 7000 | 22140 | 7000 | 340 |
| MS2 | BMS | total | IGH | 7000 | 24743 | 13000 | 1834 |
| MS2 | BMS | total | IGK | 7000 | 22150 | 13000 | 2211 |
| MS2 | BMS | total | IGL | 7000 | 10983 | 7000 | 375 |
| MS3 | BMS | Breg | IGH | 6600 | 45826 | 13000 | 5878 |
| MS3 | BMS | Breg | IGK | 6600 | 29487 | 13000 | 4362 |
| MS3 | BMS | Breg | IGL | 6600 | 23214 | 7000 | 1186 |
| MS3 | BMS | total | IGH | 6600 | 87736 | 13000 | 5602 |
| MS3 | BMS | total | IGK | 6600 | 50916 | 13000 | 5654 |
| MS3 | BMS | total | IGL | 6600 | 25926 | 7000 | 941 |
| MS4 | BMS | Breg | IGH | 23000 | 102510 | 13000 | 8835 |
| MS4 | BMS | Breg | IGK | 23000 | 48221 | 13000 | 2819 |
| MS4 | BMS | Breg | IGL | 23000 | 40365 | 7000 | 909 |
| MS4 | BMS | total | IGH | 23000 | 151702 | 13000 | 8418 |
| MS4 | BMS | total | IGK | 23000 | 83637 | 13000 | 5724 |
| MS4 | BMS | total | IGL | 23000 | 63251 | 7000 | 1384 |
| MS5 | HAMS | Breg | IGH | 15000 | 32739 | 13000 | 6244 |
| MS5 | HAMS | Breg | IGK | 15000 | 30986 | 13000 | 4739 |
| MS5 | HAMS | Breg | IGL | 15000 | 13797 | 7000 | 716 |
| MS5 | HAMS | total | IGH | 15000 | 19166 | 13000 | 1365 |
| MS5 | HAMS | total | IGK | 15000 | 13419 | 13000 | 1012 |
| MS5 | HAMS | total | IGL | 15000 | 11792 | 7000 | 458 |
| MS6 | HAMS | Breg | IGH | 12000 | 27774 | 13000 | 1051 |
| MS6 | HAMS | Breg | IGK | 12000 | 16024 | 13000 | 2505 |
| MS6 | HAMS | Breg | IGL | 12000 | 26040 | 7000 | 309 |
| MS6 | HAMS | total | IGH | 12000 | 28761 | 13000 | 2303 |
| MS6 | HAMS | total | IGK | 12000 | 27199 | 13000 | 3538 |
| MS6 | HAMS | total | IGL | 12000 | 19641 | 7000 | 531 |
| MS7 | HAMS | Breg | IGH | 12000 | 26028 | 13000 | 5508 |
| MS7 | HAMS | Breg | IGK | 12000 | 8182 | 13000 | 904 |
| MS7 | HAMS | Breg | IGL | 12000 | 7253 | 7000 | NA* |
| MS7 | HAMS | total | IGH | 12000 | 17128 | 13000 | 2903 |
| MS7 | HAMS | total | IGK | 12000 | 18665 | 13000 | 5095 |
| MS7 | HAMS | total | IGL | 12000 | 7623 | 7000 | 779 |
| MS8 | HAMS | Breg | IGH | 12000 | 29698 | 13000 | 5234 |
| MS8 | HAMS | Breg | IGK | 12000 | 35599 | 13000 | 3499 |
| MS8 | HAMS | Breg | IGL | 12000 | 24586 | 7000 | 623 |
| MS8 | HAMS | total | IGH | 12000 | 13606 | 13000 | 4611 |
| MS8 | HAMS | total | IGK | 12000 | 28416 | 13000 | 4323 |
| MS8 | HAMS | total | IGL | 12000 | 11499 | 7000 | 1045 |
| MS9 | HAMS | Breg | IGH | 5000 | 18262 | 13000 | 2511 |
| MS9 | HAMS | Breg | IGK | 5000 | 24363 | 13000 | 2731 |
| MS9 | HAMS | Breg | IGL | 5000 | 21459 | 7000 | 353 |
| MS9 | HAMS | total | IGH | 5000 | 14198 | 13000 | 2211 |
| MS9 | HAMS | total | IGK | 5000 | 24584 | 13000 | 3193 |
| MS9 | HAMS | total | IGL | 5000 | 7361 | 7000 | 618 |
|  |  |  |  |  |  |  |  |
| *samples were excluded due to low raw IG-containing reads number  HAMS - highly active MS; BMS - benign MS; HD – healthy donors | | | | | |  |  |

**Figure S1.** Distribution of CDR3 amino acid length frequencies for (A) IGK and (B) IGL clonotypes. Each boxplot represents the distribution of frequencies of clonotypes with given CDR3 length between corresponding repertoires. Rare clonotypes with CDR3 length <7 a.a. and >16 a.a. (for IGK) or >17 a.a. (for IGL) were excluded.

**Figure S2.** Correlation between age and the frequency of CD27^+^ B cells among CD19^+^CD24^high^CD38^high^ tBreg. MS – patients with multiple sclerosis. HD – healthy donors.

**Figure S3.** Relative proportion of transitional T1 and T2 subsets differentiated on the basis of (**A**) the level of CD24 and CD38 expression (**Figure 4**, flow cytometry gating strategy) or (**B**) IgD expression - T2(CD24^high^CD38^high^IgD^+^)/T1(CD24^high^CD38^high^IgD^low/-^). MS – patients with multiple sclerosis. HD – healthy donors. Mean±SD are indicated. Statistical significance of the differences between donor groups was assessed using Mann-Whitney test.
